# Assessing Copper-Alternative Products for the Control of Pre- and Postharvest Citrus Anthracnose

**DOI:** 10.3390/plants12040904

**Published:** 2023-02-16

**Authors:** Monia Federica Lombardo, Salvina Panebianco, Antonino Azzaro, Vittoria Catara, Gabriella Cirvilleri

**Affiliations:** Department of Agriculture, Food and Environment, University of Catania, Via Santa Sofia 100, 95123 Catania, Italy

**Keywords:** *Colletotrichum* spp., copper-alternative products, biocontrol, citrus, disease control

## Abstract

Citrus production is worldwide threatened by *Colletotrichum* spp., causal agents of pre- and postharvest anthracnose. The recent limitation on the use of copper-based antimicrobials, due to its demonstrated noxious effect on the environment, makes the control of this pathogen difficult. Thus, alternative products able to reduce/phase out copper in organic citrus farming are needed. In this study, the efficacy of 11 commercial alternative products were evaluated in vitro, in growth chamber, in open field and in postharvest environments. In vitro, mineral fertilizers, basic substances, essential oils, plant defense stimulators and biological control agents were able to inhibit the mycelial growth with variable efficacy. On artificially infected citrus fruit, almost all tested products significantly reduced disease incidence and severity, but with lower efficacy than copper. The efficacy of mineral fertilizers-based Kiram and Vitibiosap 458 Plus, citrus essential oil-based Prev-Am Plus and chitosan-based Biorend was confirmed in open field trials, in naturally infected citrus fruits. In these trials Biorend was the best alternative product, significantly reducing disease incidence (71% DI reduction) with better results than copper (47.5%). Field treatments reduced the incidence and severity of the disease in postharvest conditions, especially in fruits field-treated three times. Overall, selected products tested in open field can represent a good alternative to copper compounds in the view of future limitation of its use.

## 1. Introduction

Citrus is one of the most widely cultivated fruit crops, growing in more than 140 countries across the world. Of all the citrus species, sweet orange [*Citrus sinensis* (L.) Osbeck] is the most important, mainly marketed and consumed as fresh fruit, juice or concentrate. The main sweet orange fruit-producing countries are China (with a total production of more than 10 million tons), Brazil (17.1 million tons), India (9.5 million tons), United States (4.8 million tons) and Europe (6 million tons). Italy is the second European sweet orange producing country after Spain, reaching a production of over 1.6 million tons [1].

Due to favorable soil and climate conditions, the Italian sweet orange production is mainly located in Sicily (southern Italy), where the European Community (EC) has identified two major orange production districts labelled as PGI (Protected Geographical Indication) and PDO (Protected Designation of Origin). The labelled products are the “blood orange of Sicily” PGI (pigmented oranges cultivated in Catania, Enna and Syracuse provinces) and the “orange of Ribera” PDO (blond oranges cultivated in Agrigento province). Approximately 70% of the Sicilian sweet orange production is represented by the pigmented blood oranges cultivar “Tarocco” with its several clones, selected to improve quality and length of production season. The popularity of cultivar “Tarocco” in domestic and international markets is the result of several positive characteristics such as the brilliant red flesh color, agreeable fragrance, large size, balanced levels of sugar and acids and high levels of antioxidant compounds including vitamin C [2].

Citrus yield is threatened by pre- and postharvest fungal diseases, which can cause heavy reductions in production and commercialization worldwide. Among these diseases, citrus anthracnose caused by *Colletotrichum* species, one of the 10 most important plant pathogenic fungi in the world [3], has become an economically important citrus disease and represents a serious threat for orange production in all growing stages, inducing various types of symptoms [4]. Citrus infections caused by *Colletotrichum* spp., for decades considered the causal agent of postharvest anthracnose [5,6], have been more recently reported in preharvest conditions on orange crops worldwide [4,7] and with increasing frequency in different Mediterranean countries, such as Italy [8,9,10,11], Tunisia [12,13], Turkey [14] and Portugal [15]. In Sicily, epidemic anthracnose infections under preharvest conditions have been reported on blood “Tarocco” oranges [9,16], including “Tarocco Scirè”, one of the most susceptible clones cultivated in Sicily. Prolonged wet/rainfall periods favor the production and dissemination of conidial masses that are deposited on fruits where they can remain saprobically or can germinate, initiating the infection process followed by the appearance of symptoms that intensify with ripening [17]. Under favorable conditions, the small, sunken brown to black lesions on citrus fruit, can evolve and merge covering large part of the fruit surface. Moreover, several *Colletotrichum* species could determine latent infections and anthracnose symptoms can appear in apparently healthy fruit during storage, thus compromising shelf-life and market value [18].

On the basis of high incidence of symptoms in field and in postharvest conditions, anthracnose caused by *Colletotrichum* spp. can be consider an emerging disease and a serious limiting factor in sweet orange production historically established in Sicily, particularly so for the susceptible clones of “Tarocco” [9]. The control of *Colletotrichum* disease is increasingly problematic, especially in organic citrus orchards where the disease management largely involves the use of copper compounds. 

The controversy about the use of copper-based fungicides in plant protection deals with toxicity to insects, soil fauna and soil microbiota [19]. Copper may induce the emergence of copper-resistant bacterial strains [20,21] and may accumulate in soil after prolonged use, up to phytotoxic concentrations [22,23]. Copper application is currently limited to 4 kg per ha per year in most European countries [24], with a maximum of 28 kg per ha over a period of 7 years, even if higher amounts are sometime applied, as recently reported [25].

Since further restrictions on its use are expected, research is needed to explore alternative compounds and application strategies less harmful for humans, animals and environment in order to reduce copper amounts or replace it completely. This represents a priority in Italy, where organic farming is highly developed compared to other European countries, and particularly in Sicily [26]. Thus, alternative products able to reduce/phase out copper in organic citrus farming are needed. Many authors have concentrated their research on alternative products able to replace or reduce the use of copper [25,27]. Nevertheless, few studies have been carried out to evaluate alternative products in citrus cultivation. Notably, to the best of our knowledge, none of several alternative formulations available to the worldwide market has been evaluated in open field against natural anthracnose infections in citrus orchards. The aim of this study was to investigate the potential activity of commercial products based on mineral fertilizers containing different concentrations of Cu, Mn and Zn, basic substances, essential oils, plant defense stimulators and biological control agents through in vitro and in vivo experiments, and to evaluate their beneficial effects in open field treatments and in postharvest environments.

## 2. Results

### 2.1. In Vitro Antifungal Activity of Chemical and Biological Alternative Products

In in vitro assays all alternative products significantly reduced (*p* < 0.05) the mycelial growth of *C. gloeosporioides* as compared with untreated control, although with variable efficacy depending on concentration. The highest growth reductions were observed at the highest concentrations tested (1% and 0.5%) after 6 days of incubation (Table 1). Vitibiosap 458 Plus, Dentamet and Prev-Am Plus were the most effective alternative products, able to completely inhibit the mycelial growth of *C. gloesporioides* at 1% and 0.5% (94–100% growth inhibition). Their efficacy was greater than that of Ossiclor (Cu oxychloride) and Idrox (Cu hydroxide). Kiram, Amylo-X, BION and Botector showed an intermediate efficacy, causing mycelial growth reductions between 54–83% at 1% and between 54–67% at 0.5% (Table 1). The remaining products (Kiram Film, Kiram AT, Biorend and Equibasic), showed good performances only when tested at concentration of 1%, providing an average reduction of mycelial growth ranging from 40 to 51%. On the whole, all alternative products poorly reduced the growth of *C. gloesporioides* when tested at concentration of 0.1%, with the exception of Prev-Am Plus and Amylo-X which showed significantly high growth reductions even at the lowest concentrations (0.1%).

### 2.2. Antifungal Activity of Selected Commercial Products in Artificially Inoculated Fruits

**Figure 1:**
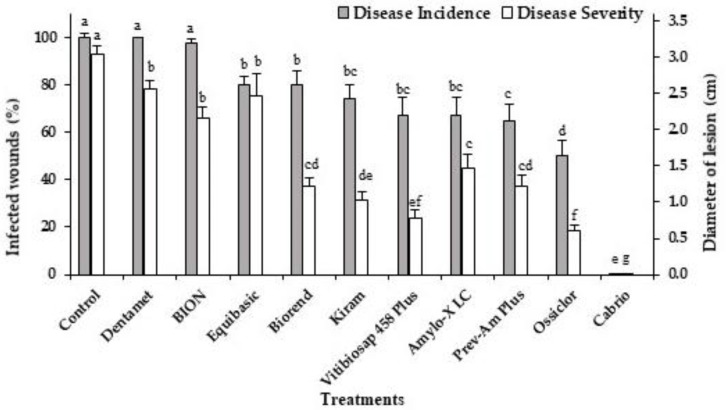
Disease incidence (infected wounds, %) and disease severity (lesion diameter, cm) on citrus fruits cv. “Tarocco Scirè” wounded, treated with alternative products, Ossiclor and Cabrio (1%) and artificially inoculated with *C. gloeosporioides*. Water-treated fruits were used as a control. Data were recorded after six days of incubation at 25 ± 1 °C and 95–98% RH. Bars represent the standard error of the mean (SEM). For each parameter, columns with different letters are statistically different according to Fisher’s least significance difference test (LSD) at *p*-value < 0.05. Data are the means of two trials.

Several alternative products significantly (*p* < 0.05) reduced disease incidence (DI, see Section 4 for a definition of this parameter) on orange cv. “Tarocco Scirè” artificially inoculated with *C. gloeosporioides,* although to a modest extent (20–35% DI reduction, not always significantly different among them) when compared to untreated control (Figure 1). Prev-Am Plus exhibited the best performances, since it was able to reduce DI to values significantly lower than in the control, although not significantly better than those recorded for Kiram, Vitibiosap 458 Plus and Amylo-X (Figure 1).

All alternative products tested significantly (*p* < 0.05) reduced disease severity (DS, see Section 4 for a definition of this parameter) by 19–74%, with Vitibiosap 458 Plus inducing the higher DS reduction, nearly as good as that observed with Ossiclor. Good performances were also recorded for Biorend, Kiram, Amylo-X and Prev-Am Plus. On the whole, these formulations were always less effective than Ossiclor and Cabrio (Figure 1). Of these two fungicides, Cabrio was the most effective in reducing DI and DS values. 

### 2.3. Antifungal Activity of Selected Commercial Products in the Field

**Figure 2:**
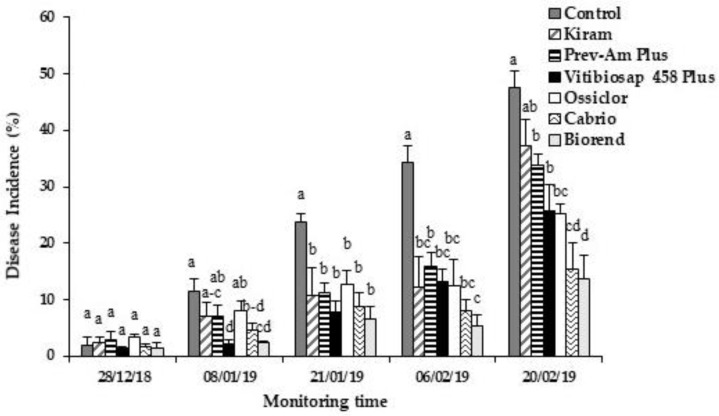
Time progression of natural anthracnose symptoms on citrus fruits cv. “Tarocco Sciré” treated in the field with alternative products, Ossiclor or Cabrio. Field treatments were carried out three times (28 December 2018; 15 January 2019; 6 February 2019). Disease incidence was expressed as percentage of symptomatic fruits/treatment. Bars represent the standard error of the mean (SEM). Columns with different letters are statistically different according to Fisher’s least significance difference test (LSD) at *p*-value < 0.05.

Selected commercial products (Vitibiosap 458 Plus, Kiram, Biorend, Prev-Am Plus) applied in open field three times from December 2018 to February 2019, significantly (*p* < 0.05) reduced the natural infections caused by *Colletotrichum* spp. in orange fruits (Figure 2). Biorend displayed the best performance, reducing the DI by 71% compared to 47.5% obtained with Ossiclor (data referred to the last monitoring time). Similarly, Vitibiosap 458 Plus significantly reduced DI (46% DI reduction), thus being comparable to Ossiclor, whereas Kiram and Prev-Am Plus showed lower efficacy (DI reduction 22% and 29%, respectively). 

**Figure 3:**
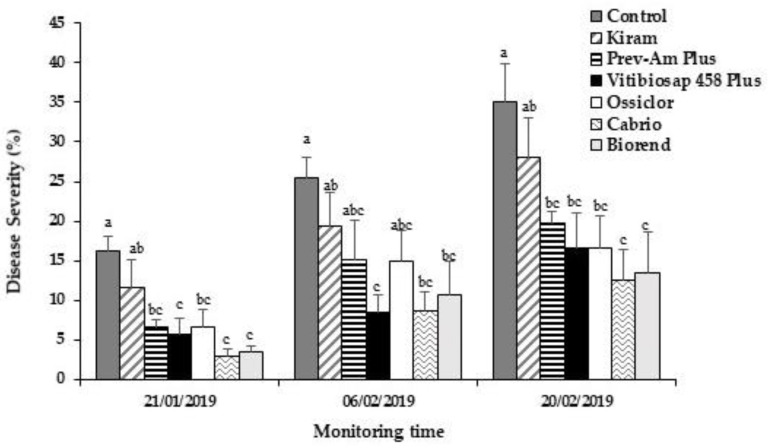
Time progression of disease severity on citrus fruit cv. “Tarocco Sciré” treated in the field with different alternative products, Ossiclor or Cabrio. Disease severity (DS%) was expressed as rating scale (see Section 4.5). Bars represent the standard error of the mean (SEM). Columns with different letters are statistically different according to Fisher’s least significance difference test (LSD) at *p*-value < 0.05.

All treatments, with the exception of Kiram, significantly reduced DS (44–62% DS reduction) if compared to the untreated control, with values similar to those observed with Ossiclor (53% DS reduction) and Cabrio (64% DS reduction) (Figure 3). 

### 2.4. Climate Data

**Figure 4:**
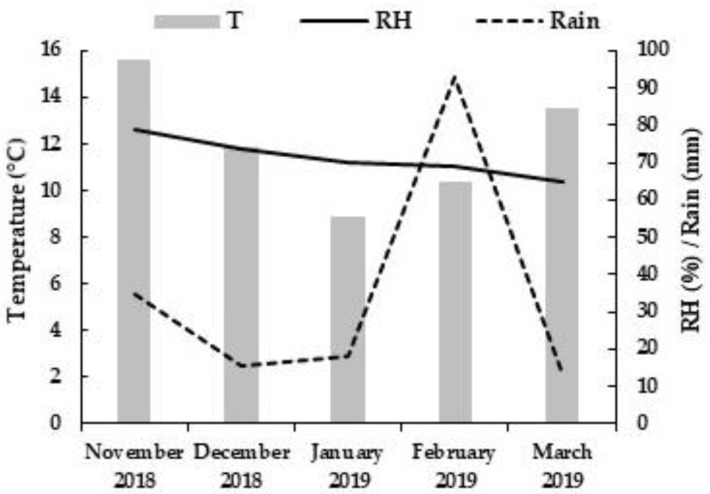
Climate data from March 2018 to March 2019, by the weather station of Francofonte (CT), Sicily. T = Temperature; RH = Relative humidity.

Climatic conditions favorable to anthracnose were recorded in the 2018/2019 season. Figure 4 reports the mean monthly values of air temperature, relative humidity and rain from 1 November 2018 up to 31 March 2019. The infection process was favored by heavy winds and rains that occurred on January–February 2019, thus resulting in higher disease pressures for the development of anthracnose symptoms during the second and third monitoring.

### 2.5. Efficacy of Field Treatments in Postharvest Environments

All field treatments significantly (*p* < 0.05) reduced DI and DS after the storage periods (6 °C for 20 days and then at 20 °C for 10 days) compared to untreated controls. 

**Figure 5:**
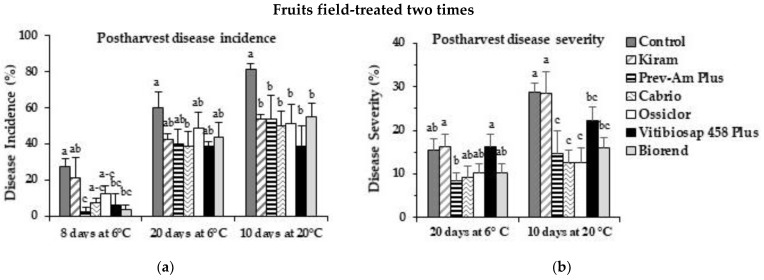

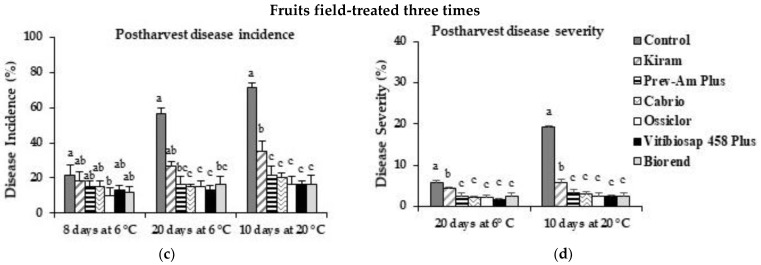
Disease incidence (%) and disease severity (%) on citrus fruits cv. “Tarocco Scirè” field-treated two times (**a**,**b**) and three times (**c**,**d**), after storage at 6 °C for 20 days and at 20 °C for 10 days. Bars represent the standard error of the mean (SEM). Columns with different letters are statistically different according to Fisher’s least significance difference test (LSD) at *p*-value < 0.05.

In fruits field-treated two times, DI (Figure 5a) was significantly (*p* < 0.05) reduced (DI reduction 32.3% to 52.3%) (data at the end of storage periods), with values similar to those recorded for Ossiclor and Cabrio. Similarly, DS (Figure 5b) was reduced by all products, except Kiram (DS reduction 22.6% to 48.7%). 

Likewise, DI (Figure 5c) was significantly reduced in fruits field-treated three times (DI reduction 51.1% to 76.7%) and all products, except Kiram, showed an efficacy significantly (*p* < 0.05) similar to Ossiclor and Cabrio. Similarly, DS was reduced by all products (DS reduction 83.3% to 87.2%) (Figure 5d), with effects comparable to those observed with Ossiclor and Cabrio. On the whole, the third field treatment significantly improved the overall results compared with only two treatments (Figure 5). Most important, pre-harvest treatments significantly protected the fruits from anthracnose with all tested compounds except Kiram. 

## 3. Discussion

Citrus anthracnose is a severe disease that in recent years has become responsible for heavy losses in the production of “Tarocco” blood oranges in Sicily [9]. Only few fungicides and Cu compounds are registered to control *Colletotrichum* spp. on citrus crop in Italy. The increasing resistance of pathogens to fungicides and the growing public concerns on fungicide residues in food and environment have made the search for alternative products urgent. Moreover, the new laws that progressively limit the use of copper in European countries [25] have made increasingly difficult the control of pre- and postharvest diseases in the areas managed with organic methods, making the search for alternative products to copper even more urgent. 

Recently, the use of non-chemical treatments has gained attention as approach of choice in integrated strategies to control pre- and postharvest fruit anthracnose caused by *Colletotrichum* spp. in different plant species, including avocado, dragon fruit, papaya, banana, mango [28,29,30,31,32], whereas only few alternative products have been evaluated so far for the control of anthracnose in citrus [33,34,35].

Among mineral fertilizers tested in this study, Dentamet (based on zinc and copper complexed with citric-acid hydracids) has been recently evaluated in laboratory and in the field for the control of *Xylella fastidiosa* subsp. *pauca* in olive [36,37], *Plenodomus tracheiphilus* in citrus [38] and *Xanthomonas euvesicatoria* pv. *perforans* in tomato [39]. Mineral nutrients are directly involved in plant protection, since the nutritional status affects plant’s susceptibility or resistance to pathogens and pests through the activation of enzymes that produce defense metabolites [40]. With regard to micronutrients, involvement in plant defense has been predominantly documented for Cu, Mn and Zn [41]. In addition to the well-known efficacy of copper in plant defense, manganese participates in the production of lignin, suberin, phenolic compounds and other plant defense mechanisms, while zinc is involved in membrane protection against oxidative damages and in the induction of pathogenesis-related proteins [41,42]. In the in vitro assays carried out in the present work, the mineral fertilizers Vitibiosap 458 Plus, Dentamet and Kiram, containing different levels of Cu (2–6%) Zn (1–4%) and Mn (0.1%), showed a direct antimicrobial effect, completely inhibiting or significantly reducing the mycelial growth of *C. gloesporioides* with efficacy higher or similar to that observed with copper compounds. In our study, the antifungal activity of Vitibiosap 458 Plus and Kiram was also confirmed on *C. gloesporioides*-inoculated fruits, on which a significant reduction of disease incidence and severity was observed. The same good disease control was achieved when Vitibiosap 458 Plus was applied in open field to control anthracnose in naturally infected citrus fruits. On “Tarocco Scirè”, a high susceptible clone of “Tarocco”, Vitibiosap 458 Plus reduced the disease incidence much as observed with copper oxychloride (46% and 47.5% DI reduction, respectively). Accordingly, a direct antimicrobial activity of Zn- and/or Mn-containing compounds has been a reported towards *Fusarium verticilloides* [43], *Phytophthora nicotianae* [44] and *Lasiodiplodia theobromae* [45]. Moreover, the ability of compounds based on Zn or Mn to induce metabolic changes in the host plants, thus allowing to reach significant reductions of disease symptoms, has also been reported in greenhouse and open field conditions in coffee seedlings [46], in cowpea roots [47] and in olive groves [37]. 

As for mineral fertilizers, essential oils (EOs) from various plant species have been employed in integrated strategies to control pre- and postharvest diseases caused by several plant pathogens, including *Colletotrichum* spp. [48,49]. Among EOs, citrus essential oils (CEOs) have attracted attention as antifungal agents owing to their antimicrobial properties against a broad-spectrum of plant pathogens [50,51]. In this study, the effectiveness of a commercial formulation based on sweet orange essential oil, namely Prev-Am Plus, was tested. This product is authorized in Italy, both in greenhouse and in open field, to control several insect pests and fungal pathogens in a diversity of crops including olive trees, vines and citrus fruits. In in vitro assays, Prev-Am Plus displayed a remarkable antifungal activity at all tested concentrations, and the rates of growth reduction (87–99% at 1, 0.5 and 0.1% commercial product) were significantly similar or higher than with copper compounds (71–94%). Our results were in agreement with those of Abd-Alla and Haggag [52], who reported 70–100% of *C. gloeosporioides* growth inhibition with pure grade orange essential oils at concentrations ranging from 100–150 µg/mL. The antifungal activity of CEOs has also been tested against *C. gloeosporioides* on artificially inoculated papaya and mango fruit [52,53] and showed significant reductions of both incidence and severity. Similarly, in our studies, CEOs-based Prev-Am Plus significantly reduced DI and DS of anthracnose by *C. gloeosporioides* in artificially inoculated orange fruit, ranking best among all alternative formulations tested. To the best of our knowledge, this is the first study that demonstrated the efficacy in open field of CEOs-based Prev-Am Plus in the control of anthracnose by *Colletotrichum* in orange fruits. 

Furthermore, in this work two basic substances, Biorend (chitosan) and Equibasic (equisetum) have been studied. Chitosan, a natural biodegradable chitin-derivative, is the most studied product thanks to its well-known direct fungicidal activity and indirect activity as elicitor of resistance to numerous pre- and postharvest fungal diseases [54,55,56,57,58,59], including *Colletotrichum* spp. [32,33,34,35]. In contrast, the potential of equisetum (field horsetail) in the control of plant diseases has been investigated less extensively. It is known to have preventive effects on fungal diseases, attributable to the high percentage of silica [60,61] and antifungal effects linked to its high concentration of flavonoids and phenols [62]. To date, there are some reports documenting its efficacy in the control of plant pathogens such as *Plamopara viticola* [63,64], *Alternaria solani* [65], *Phytophthora infestans*, *Puccinia triticina* [66] and grapevine trunk pathogens [67]. In in vitro assays carried out in this work, both Biorend (chitosan) and Equibasic (equisetum) significantly inhibited the growth of *C. gloeosporioides* at all the tested concentrations, with best results observed at 1% of commercial product. The in vitro inhibitory effect of chitosan against *Colletotrichum* spp. observed in the present study has already been demonstrated in several previous studies [35,68,69,70], in some of which structural changes in the conidial morphology [68] and spore germination [35] have been reported. The in vitro inhibitory activity of equisetum against *C. gloeosporioides* is here reported for the first time.

Biorend and Equibasic tested on *C. gloeosporioides*-inoculated citrus fruits in growth chamber significantly reduced disease incidence, and Biorend showed higher disease severity reduction. Considering the potential of chitosan as an antifungal agent, we extended our investigation in open field on naturally infected “Tarocco Scirè”, where it proved to be the best alternative product, reducing disease incidence by 71%, with values significantly higher than copper oxychloride (47%). On the whole, Biorend seemed to be more efficient when applied to plants in the field or to harvested fruits in growth chamber than in tests on agar plates. These results are in agreement with those reported in a recently published meta-analysis which summarized studies related to the effects of chitosan on disease control and on its antimicrobial and eliciting abilities [55]. The analysis involved several fungal pathogens and revealed that, in the in vitro assays, *Colletotrichum* spp. is the least sensitive pathogen to chitosan. Instead, in *in- planta* tests, chitosan showed the highest effectiveness against anthracnose, clearly showing that the outcome of chitosan-pathogens interactions may vary with plant species. 

Among the chemical compounds able to induce plant resistance, the most deeply investigated are those interfering with the salicylic acid (SA) pathway, such as acibenzolar-S-methyl (ASM), developed and marketed in Europe with the label BION and in the United States with the label ACTIGARD [71]. ASM has proved to be effective in the control of diseases caused by viral, bacterial and fungal pathogens in different plant species [72,73,74,75]. In this work, BION has been tested for the first time for its antifungal activity in vitro and in inoculated fruits against *C. gloeosporioides* in growth chamber trials. In vitro, BION exhibited a direct antimicrobial activity and significantly reduced the growth of *C. gloeosporioides* at the highest concentration tested (58% and 52% at 1% and 0.5%, respectively). Accordingly, previous studies demonstrated that similar concentrations of BION directly inhibited in vitro mycelial growth of *Penicillium digitatum* [57]. In contrast, BION caused no significant reduction of disease incidence and only a slight reduction of disease severity in artificially inoculated “Tarocco Scirè” fruits. The lack of efficacy of BION against *C. gloeosporioides* in artificially inoculated citrus fruits is in agreement with the results previously reported by Panebianco et al. [57] in “Tarocco Scirè” fruits artificially inoculated with *P. digitatum*. Furthermore, plant activator such as BION need at least a few days to induce resistance whereas in our experiment, due to a very short time, the effect of inducing resistance did not appear.

Among biocontrol agents (BCAs), two commercially available products (Amylo-X and Botector) showed antimicrobial activity in in vitro tests against *C. gloeosporioides* (59% and 54% growth reduction, respectively), and the antimicrobial activity was confirmed for Amylo-X when applied to artificially inoculated “Tarocco Scirè” fruits. There are few studies on the use of BCAs for the control of *Colletotrichum* spp. in citrus fruits. *Bacillus subtilis* showed efficacy under field conditions in controlling postbloom fruit drop (PFD) caused by *C. abscissum* (previously reported as *C. acutatum)* or *C. gloeosporioides* [76,77] and *S. cerevisiae* isolates showed to be effective in controlling *C. abscissum* both preventively and curatively in preharvest experiments [78]. On the whole, biocontrol agents are rarely employed in open field, because they are often poorly resistant to environmental conditions encountered in the field, which negatively influence survival and antagonistic behavior. Furthermore, for perennial crops such as citrus fruits, where the inoculum can survive from one year to the next, an effective biological control requires a preventive and curative strategies, possibly with products capable of acting directly on the pathogen and indirectly by activating host’s natural defenses.

Finally, the efficacy of field treatments in controlling *Colletotrichum* spp. under postharvest conditions was evaluated on oranges stored at 6 °C for 20 days and then at 20 °C for 10 days. The results achieved in postharvest environments highlighted the ability of all products to protect citrus fruits from *Colletotrichum* infections during their storage for at least one month. The highest protection against postharvest anthracnose was achieved with three-times field applications of Vitibiosap 458 Plus, Prev-Am Plus and Biorend, and their efficacy was similar to that of Ossiclor and Cabrio, used as control. With regard to chitosan and its derivatives, our results are in agreement with previous works [30,31], in which preharvest applications of oligochitosan effectively enhanced the postharvest resistance of navel orange artificially inoculated with *C. gloeosporioides*. In the same studies, the authors showed that preharvest application of oligochitosan enhanced the activity of defensive enzymes, increased the level of hydroxyproline-rich protein and delayed protopectin degradation, suggesting that structural and biochemical mechanisms of resistance were induced in preharvest and persisted during the subsequent storage period. 

On the whole, Vitibiosap 458 Plus, Biorend and Prev-Am Plus might represent interesting substitutes for copper compounds against *Colletotrichum* spp. in citrus orchards. Their good efficacy, here evaluated in laboratory conditions and validated in open field for the first time, strongly encourage subsequent trials to explore their potential for large-scale use in citrus organic orchards. Disease control was higher in open field than in the harvested fruits artificially inoculated with the pathogen, as in the latter the compounds strongly inhibited only the severity but not the incidence of the disease. Thus, the present study underlines the importance of considering the multiple trophic levels (bioproduct- pathogen- host-carpospheric microbioma- environment) involved in plant/pathogen interactions. 

## 4. Materials and Methods

### 4.1. Colletotrichum gloeosporioides Isolate

*C. gloeosporioides* isolate, obtained from citrus fruit showing typical symptoms of disease and previously identified, belongs to Di3A collection (Dipartimento di Agricoltura Alimentazione e Ambiente, University of Catania, Italy) [16]. The isolate, kept in Potato Dextrose Agar (PDA, Oxoid, Basingstoke Hempshire, UK) at 4 °C, was used in the in vitro and in growth chamber assays. Subcultures were grown for 10 days at 25 °C in the dark before each assay. 

### 4.2. Chemical and Biological Alternative Commercial Products

Alternative commercial products used in in vitro and in in vivo assays were (1) five mineral fertilizers containing different concentrations of Cu, Mn and Zn [(Vitibiosap^®^ 458 Plus (NDG Group; Cu 3.5%, Zn 1%; water-soluble liquid); Kiram (Agriges; Cu total, 6.0%, chelated Cu, 0.1%, soluble Cu, 0.1%, Mn, 0.1%, chelated Mn, 0.1%, soluble Zn, 0.1%, chelated Zn, 0.1; water-soluble); Kiram AT (Agriges; total Cu, 1.8%, soluble Cu, 0.4%, chelated Cu, 0,4%, water-soluble); Kiram Film (Agriges; total B, 0.2%, soluble Mn, 0.1%, chelated Mn, 0.1%, total Zn, 1.7%, soluble Zn, 0.2%, chelated Zn, 0.2%; water-soluble); Dentamet^®^ (Diagro; Cu, 2.0%, Zn, 4.0%; water-soluble)]; (2) one plant defence stimulator [BION^®^ 50 WG (Syngenta; 50% Acibenzolar-S-methyl; water dispersible granules)]; (3) two basic substances [Equibasic^®^ (Idainature; 0.2% extract of *Equisetum arvense* L.; water-soluble liquid) and Biorend^®^ (Bioplanet; 1.9% chitosan hydrochloride; water-soluble liquid)]; (4) one citrus essential oils [Prev-am Plus^®^ (Nufarm; 5.8% sweet orange essential oil; water-soluble liquid)]; (5) two biological control agents [Amylo-X^®^ LC (Biogard; 5% *Bacillus amyloliquefaciens* subsp. *plantarum* D747; liquid formulations) and Botector^®^ New (Manica; *Aureobasidium pullulans* DSM14940-14941; water dispersible granules)]. Copper oxychloride (Ossiclor 35 WG, Manica; 35% active ingredient (a.i.); water dispersible granules), copper hydroxide (Idrox 20 WG, Manica; 20% a.i.; water dispersible granules) and pyraclostrobin (Cabrio^®^ WG, Basf; 20% a.i., water dispersible granules) were used as control. 

### 4.3. In Vitro Antifungal Activity of Chemical and Biological Alternative Commercial Products

The 11 alternative products chosen for this study were tested against *C. gloeosporioides* on PDA amended with three decreasing concentrations of products. Stock solutions were prepared in sterile distilled water (SDW). Specifically, autoclaved PDA media, cooled at 45–50 °C, were amended with appropriate volumes of stock solutions to obtain the following final concentrations: 1%, 0.5%, 0.1% (*w*/*v*). Four plates, for each concentration, were used. Mycelial plugs (0.5 cm-diameter), axenically taken from the edge of *C. gloeosporioides* colonies by a sterile cork-borer, were placed centrally on amended and solidified PDA plates. Control samples consisted of non-amended PDA plates inoculated with the mycelial plug. PDA plates amended with copper oxychloride (Ossiclor) and copper hydroxide (Idrox) were used as chemical control. The colony growth (average of two orthogonal diameters, cm) was recorded after 6 and 10 days of incubation at 25 ± 1 °C in the dark. Each experiment was conducted twice and included three replicates per treatment. 

### 4.4. In Vivo Antifungal Activity of Selected Alternative Commercial Products in Artificially Inoculated Fruit

Eight alternative commercial products (Vitibiosap 458 Plus, Kiram, Dentamet, BION, Equibasic, Biorend, Prev-Am Plus and Amylo-X) were selected on the basis of the results achieved in in vitro assays and tested in artificially inoculated citrus fruits. Orange fruits cultivar “Tarocco Scirè” were harvested at physiological maturity in a conventional farm located in Syracuse area (south-east Sicily). Fruits, uniform in size, colour, ripeness and free of defects or injuries, were used immediately or held at 4 °C for no longer than one week before use. The selected fruits were washed with tap water, surface-sterilized by dipping for 2 min in a 2% sodium hypochlorite solution, rinsed with SDW and then left to dry at room temperature. To produce inoculum, colony surface of *C. gloeosporioides* isolate grown on PDA for 10 days at 25 ± 1 °C was washed with 10 mL of a 0.05% (*v*/*v*) Tween 80 solution in SDW, the suspension was filtered through a double layer of sterile gauze and the number of conidia was adjusted to a final concentration of 10^5^ conidia/mL by an hemocytometer. Citrus fruits were wounded with a sterile nail (3 mm wide ×3 mm depth) at two opposite points in the area between the peduncle and the equatorial axis. Next, 100 μL of product solutions (1% in SDW) were pipetted into each wound and, after drying, the wounds were inoculated with drops (20 μL) of conidial suspension of *C. gloeosporioides* (10^5^ conidia/mL). Wounds treated with SDW or with Ossiclor or with Cabrio served as control. Fruits were incubated in sterile plastic boxes (20 fruits per box) containing wet paper towels. Plastic boxes were put into plastic bags and incubated at 25 °C and 95% RH for 15 days. Each treatment had two replicates, consisting of 20 fruits for each replicate (40 fruits/80 wound per treatment). Data concerning DI (% of infected wounds) and DS (diameter of infected area around the wounds, cm) were calculated as the average of each replicate 15 days post treatment. The experiments were repeated twice.

### 4.5. Antifungal Activity of Selected Commercial Products under Field Conditions

**Figure 6:**
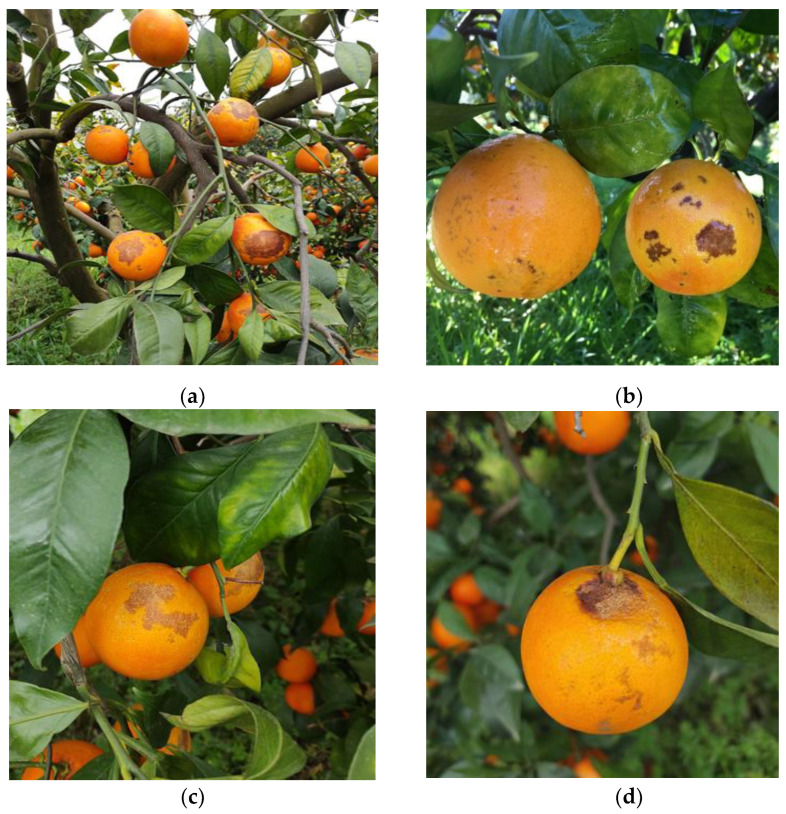
Anthracnose disease symptoms on naturally infected “Tarocco Sciré” fruits: severe anthracnose fruit spot (**a**); small to large lesions (**b**,**c**); necrotic lesions at the stem-end of fruit (**d**).

Field experiments were carried out from December 2018 to February 2019 to evaluate the efficacy of four alternative products (Vitibiosap 458 Plus, Kiram, Biorend, Prev-Am Plus) against natural infections caused by *Colletotrichum* spp. (Figure 6a–d). Alternative products were selected among the products that in previous trials had significantly reduced both DI and DS, and/or authorised for use in citrus orchards and compatible with organic farming regulations. Experiments were carried out on *C. sinensis* cultivar “Tarocco Scirè” in an orchard located at Pedagaggi (37°11′ 30.27″ N, 14°55′ 37.52″ E, altitude 307 m a.s.l.—Syracuse province). This experimental site had a history of severe attacks of citrus anthracnose on high susceptible “Tarocco Scirè” cultivar [11]. The trial was arranged in a randomized complete block design with 4 replicates of 4 plants for each treatment (16 plants per treatment). Treatments consisted of application of Biorend (300 mL/hL), Prev-Am Plus (400 mL/hL), Kiram (250 mL/hL) and Vitibiosap 458 Plus (125 g/hL)]. Ossiclor (350 g/hL) and Cabrio (56.25 g/hL) were used as standard fungicide. Control citrus plants were untreated. Treatments were sequentially performed three times within the period from December 2018 to February 2019 (28 December 2018; 15 January 2019; 6 February 2019) according to label instructions by motorized backpack mist blower, approximately 1500 L ha^−1^. The first application of products was carried out on asymptomatic fruits. All treatments were applied early in the morning and each tree was sprayed to run-off. Disease incidence was visually assessed five times during the trial, calculating the percentage (%) of infected fruits within the four replicates for each treatment (50 fruits/plant, 4 plants/replicate, randomly sampled from the 4 cardinal orientations of each plant). Disease severity was recorded three times as the percentage of symptomatic fruit area classified according to a six classes rating scale of damage [29] as follows: class 0 = no symptoms, class 1 = a few scattered lesions covering 1–5% of the fruit surface; class 2 = a few scattered lesions covering 6–10% of the fruit surface; class 3 = lesions covering 11–25% of the fruit surface; class 4 = extensive lesions covering 26–50% of the fruit surface; class 5 = severe lesions covering > 50% of the fruit surface. From the disease rating, disease severity was expressed in percentage (%) and calculated according to the following formula:

(1) $$\mathrm{DS}\;(\%)=\frac{\sum \left(\mathrm{class}\;\mathrm{frequency}\;\times \;\mathrm{score}\;\mathrm{of}\;\mathrm{rating}\;\mathrm{class}\right)}{\left(\mathrm{total}\;\mathrm{number}\;\mathrm{of}\;\mathrm{fruit})\;\times \;(\mathrm{maximal}\;\mathrm{disease}\;\mathrm{index}\right)}\;\times \;100$$

### 4.6. Climate Data

The weather parameters, i.e., average temperature (°C), relative humidity (%) and rainfall (mm) were obtained from the data provided by the weather stations of Francofonte (Syracuse province) of Servizio Informativo Agrometeorologico Siciliano (SIAS), Sicily region. 

### 4.7. Effect of Field Treatments on Postharvest Environments

The efficacy of field treatments on controlling infections of *Colletotrichum* spp. during postharvest was evaluated. Apparently healthy fruits were harvested after the second (21 January 2019) and the third (13 February 2019) field treatment. Fruits (80 fruits per treatment-20 fruits/replicate) were placed in sterile plastic boxes (20 fruit per box) with a corrugated paper at the bottom and covered with a paper and plastic sheet. Fruit boxes were stored 20 days under controlled temperature in climatic cell (6 ± 1 °C and 90% RH) (cold storage) followed by a shelf-life evaluation during 10 days at 20 ± 1 °C. Disease incidence (percentage of infected fruits within the four replicates of each treatment) and disease severity (percentage of symptomatic fruit area according to a six classes scale of damage) were evaluated after 8 and 20 days at 6 °C and after 10 days at 20 °C, following the procedure described above (Section 4.5).

### 4.8. Statistical Analysis

All statistical analyses were performed using Minitab software (Minitab, version 19). Mean data were subjected to one-way analysis of variance (ANOVA) and tested for significant differences among treatments by Fisher’s least significant differences (LSD). A *p*-value of <0.05 was considered statistically significant. Data for disease incidence were transformed using arcsine (sin–1 square root x) prior to statistical analysis to make their distribution normal.

## 5. Conclusions

To our knowledge, this study provides evidence for the first time of the good efficacy of some commercially available alternative products against the pre- and postharvest citrus disease caused by *Colletotrichum* spp., in the perspective of replacing or reducing traditional copper treatments and safeguard the quality of Sicilian sweet orange production. Despite high disease pressure conditions, field applications of alternative products significantly reduced the incidence and severity of citrus anthracnose on a high susceptible citrus cultivar such as the “Tarocco Scirè” both in the field and during storage. The good results obtained in this study provide useful information for the field management in organic citrus orchards. These encouraging data must be considered preliminary and will be verified in future field trials to assess the effectiveness of alternative commercial products in different soils and climatic conditions in other citrus species.

## Figures and Tables

**Table 1:** *Colletotrichum gloeosporioides* colony diameter (cm) on PDA medium amended with different potential fungicides at different concentrations (1%, 0.5%, 0.1%). Plates were incubated at 25 ± 1 °C in the dark for 6 days.

Class of Products		Colony Diameter (cm) ± SEM *		
	Products	1%	0.5%	0.1%
	Control	8.5 ± 0.0 a	8.5 ± 0.0 a	8.5 ± 0.0 a
Mineral fertilizers	Vitibiosap 458 Plus^®^	0.0 ± 0.0 h	0.0 ± 0.0 i	6.0 ± 0.2 cd
	Dentamet^®^	0.0 ± 0.0 h	0.5 ± 0.0 i	6.0 ± 0.3 cd
	Kiram^®^	1.4 ± 0.1 g	2.8 ± 0.2 fg	8.2 ± 0.1 ab
	Kiram Film^®^	4.1 ± 0.1 d	5.4 ± 0.1 d	6.2 ± 0.1 cd
	Kiram AT^®^	4.5 ± 0.0 cd	7.1 ± 0.1 b	7.7 ± 0.1 b
Plant defence stimulator	BION^®^ (Acibenzolar-S-methyl)	3.5 ± 0.2 e	4.1 ± 0.2 e	5.4 ± 0.2 d
Basic substances	Biorend^®^ (Chitosan hydrochloride)	4.9 ± 0.1 bc	6.3 ± 0.1 c	7.6 ± 0.2 b
	Equibasic^®^ (Equisetum)	5.1 ± 0.1 b	5.7 ± 0.1 d	6.7 ± 0.1 c
Essential oil	Prev-Am Plus^®^ (Sweet orange essential oil)	0.1 ± 0.0 h	0.4 ± 0.1 i	1.1 ± 0.4 f
Biological control agents	Amylo-X^®^ LC (*B. amyloliquefacines* D747)	2.6 ± 0.3 f	3.0 ± 0.2 f	3.0 ± 0.2 e
	Botector^®^ (*A. pullulans* DSM14940)	3.9 ± 0.4 de	3.9 ± 0.8 e	8.3 ± 0.1 ab
Cu fungicides	Ossiclor^®^ (Copper oxychloride)	0.5 ± 0.0 h	2.4 ± 0.2 g	2.5 ± 0.2 e
	Idrox^®^ (Copper hydroxide)	1.4 ± 0.04 g	1.6 ± 0.1 h	5.5 ± 0.0 d

* For each concentration data presented as mean (±SEM) followed by the same letter indicate no difference among the tested products according to Fisher’s least significance difference test (LSD) at *p*-value < 0.05. Data are the means of two trials.

## Data Availability

The data presented in this study are available on request from the corresponding author.
